# Undertriage in geriatric trauma: insights from a multicentre cohort study

**DOI:** 10.1186/s13049-025-01432-0

**Published:** 2025-07-10

**Authors:** Daniel Anthony Koch, Lars Becker, Uwe Schweigkofler, Paul Hagebusch, Philipp Faul, Christian Waydhas, Florian Pavlu, Markus Baacke, Caspers Michael, Valentin Clemens, Helena Duesing, Matthias Froehlich, Sebastian Imach, Kai-Oliver Jensen, Christian Kleber, Annette Keß, Gerrit Matthes, Andre Nohl, Orkun Oezkurtul, Thomas Paffrath, Vera Pedersen, Kai Sprengel, Philipp Stoermann, Heiko Trentzsch, Rolf Lefering, Dan Bieler, Lisa Hackenberg, The Committee on Emergency Medicine, Intensive Care and Trauma Management (Sektion NIS) of the German Trauma Society (DGU) Germany

**Affiliations:** 1 Department of Trauma Surgery and Orthopaedics, BG Unfallklinik Frankfurt am Main, Frankfurt am Main, Germany; 2 Department of Trauma Surgery, Hand- and Reconstructive Surgery, Essen University, Essen, Germany; 3 Department of Trauma Surgery and Orthopaedics, Reconstructive Surgery, Hand Surgery and Burn Medicine, German Armed Forces Central Hospital Koblenz, Koblenz, Germany; 4 Department of Trauma Surgery, Hospital of the Merciful Brothers Trier, Trier, Germany; 5 Department of Trauma and Orthopaedic Surgery, Cologne-Merheim Medical Centre (CMMC), Cologne, Germany; 6 Department of Orthopaedic and Trauma Surgery, Dritter Orden Clinic Munich, Munich, Germany; 7 Department of Trauma, Hand and Reconstructive Surgery, University Hospital Muenster, Muenster, Germany; 8 Department of Trauma, Faculty of Medicine, University Hospital Zurich (USZ), Zurich, Switzerland; 9 Clinic and Polyclinic for Orthopaedics, Trauma Surgery and Plastic Surgery, University Hospital Leipzig AöR, Leipzig, Germany; 10 Department of Trauma and Reconstructive Surgery, Ernst von Bergmann Klinikum, Potsdam, Germany; 11 Centre for Emergency Medicine, BG Klinikum Duisburg, Duisburg, Germany; 12 Medical Director Rescue Service, Oberhausen Fire Brigade, Oberhausen, Germany; 13 Department of Trauma and Hand Surgery, Augustinerinnen Hospital, Cellitinnen- Severinsklösterchen, Cologne, Germany; 14 Department of Orthopaedics and Trauma Surgery, Musculoskeletal University Centre Munich (MUM), LMU Klinikum, Munich, Germany; 15 Faculty of Health Sciences and Medicine, University of LucerneHirslanden Clinic St. Anna, Praxis medOT, St-Anna-Str. 32, Lucerne, 6006 Switzerland; 16 Department of Trauma and Orthopaedic Surgery, University Hospital Frankfurt, Frankfurt am Main, Germany; 17 Institut für Notfallmedizin und Medizinmanagement (INM), LMU Klinikum, LMU Munich, Munich, Germany; 18 Institute for Research in Operative Medicine (IFOM), Witten/Herdecke University, Cologne, Germany; 19 Department of Orthopaedics and Trauma Surgery, University Hospital Düsseldorf, Medical School Heinrich Heine University, Duesseldorf, Germany

**Keywords:** Severely injured, Trauma team activation, Geriatric trauma, Geriatric patient, Emergency medicine, Guideline, Undertriage

## Abstract

**Background:**

With the aging population, the number of geriatric trauma patients continues to rise, posing significant challenges for emergency care and trauma management. Structured trauma team activation (TTA) protocols aim to provide timely and adequate treatment for severely injured patients. However, evidence suggests that current triage criteria may inadequately address the specific needs of geriatric patients, potentially leading to undertriage and worse outcomes.

**Methods:**

The prospective, multicentre observational cohort study analysed trauma team activation and triage practices for patients aged ≥ 70 years across 12 Level 1 trauma centres across rural and urban regions in Germany and Switzerland. Data were prospectively collected from December 2020 to February 2021, following the STROBE guidelines. Triage decisions were compared with the TAcTIC (Trauma Team Activation and Trauma/Injury Care) consensus criteria to assess undertriage and overtriage rates. Key outcomes included trauma team activation rates, injury severity, transport characteristics, and early mortality.

**Results:**

Among 3,753 trauma patients, 1,371 (36.5%) were geriatric (≥ 70 years). Trauma team activation was significantly lower in the geriatric group (15.8%) compared to younger patients (31.8%), despite similar injury severity. Post-hoc analysis revealed that 53.8% of geriatric patients requiring trauma care were undertriaged. Head injuries (47.7%) and pelvic fractures (5.7%) were more common in geriatric patients in comparison to the younger cohort. Mortality within 48 h was more than three times as high in geriatric patients (1.8% vs. 0.5%).

**Conclusion:**

A significant undertriage rate (53.8%) was identified among geriatric trauma patients, contributing to delayed care and increased mortality. Undertriage of geriatric trauma patients remains a critical issue, reflecting the insufficiency of current trauma activation protocols. Tailored triage criteria that even more consider age-related physiological differences, comorbidities, and frailty are urgently needed. Future updates to trauma guidelines should aim to reduce undertriage and improve outcomes for this vulnerable population.

**Clinical trial number:**

Not applicable

## Introduction

Demographic change with an aging society brings challenges on many levels, including the management of severely injured patients. The reasons for this seem plausible, as the older members of our society are more mobile in general and particularly in traffic. This may result in more accidents involving this population. In addition, a high level of activity in leisure time, sport and self-care can also be observed [1]. However, even minor incidents, such as “low falls” at home, tripping over a carpet edge or slipping on stairs, can result in life threatening severe injuries, highlighting the substantial injury risk associated with these seemingly trivial events.

Despite the implementation of structured, priority-oriented treatment concepts to improve the care of severely injured patients of all age groups - both prehospital (PHTLS^®^) and clinical (ATLS^®^, ATCN^®^) - there still seem to be deviations in the triage and thus the treatment of severely injured geriatric patients. 

The adapted trauma team activation criteria for initial management of severe injuries in geriatric patients over 69 years of age are critical to provide adequate and timely care. Historically, these patients may have been undertriaged due to several factors, including age-related comorbidities, diminished physiological reserves, and atypical clinical presentations of injuries.

Previous triage was often based on conventional age limits and standardized criteria that may not have adequately considered the specific needs and risks of geriatric patients. However, this approach may have changed with the introduction of the new German “Guideline on Polytrauma and Treatment of Severely Injured Patients” by the German Society for Trauma Surgery (DGU) in 2023 [2]. This guideline aims to enable improved triage and treatment of severe injuries by providing an individualized and holistic assessment of patients, considering their age-related characteristics. Similarly, the “Resources for Optimal Care of the Injured Patient” (2022) of the American College of Surgeons Committee on Trauma (ACS COT) [3] emphasizes the need for tailored trauma care for geriatric patients in the Unites States of America. The updated standards recognize that older patients have distinct physiological responses to injury, necessitating specific adjustments in trauma team activation, resuscitation protocols, and post-acute care to optimize outcomes.

In this publication, we will analyse the current practice of trauma team activation and triaging severe injuries in geriatric patients (≥ 70 years) and discuss the potential impact of the new guideline on the initial treatment in emergency room of this patient group. We will address the challenges associated with the appropriate identification and treatment of severe injuries in older patients, as well as the opportunities that could arise from improved consideration of the individual needs of this population.

## Materials and methods

### Study design

A prospective, multicentre observational cohort study was conducted across 12 trauma centers, all of which are certified as supra-regional, level 1 trauma centres-facilities providing the highest level of trauma care. Data were collected from 12 Level centers, including both urban and rural regions across Germany and Switzerland. Blunt trauma accounted for the vast majority of cases (> 95%), while penetrating injuries were rare across all centers. Given the consistent predominance of blunt trauma and the high-level trauma care provided at each site, we did not perform a detailed subgroup analysis of urban/rural variations or trauma mechanism breakdown by region.

To ensure an adequate sample size for subgroup analyses, the study aimed to include a minimum of 3,000 severely injured that were admitted to the emergency department. Based on patient volume projections, each participating centre undertook data collection over a three-month period between December 2020 and February 2021.

The study followed the “Strengthening the Reporting of Observational Studies in Epidemiology (STROBE)” guidelines and complied with the ethical principles outlined in the Declaration of Helsinki and its amendments. Approval was granted by the lead ethics committee at the University of Leipzig (reference number 060/18-ek), with subsequent approval obtained from local ethics committees at all participating centres. Written informed consent was secured from patients as early as feasible post-injury.

No study-specific interventions were performed; data collection was limited to routine clinical information.

Prehospital recommendations for trauma team activation (TTA), including both previous (2016) and updated (2023) guidelines for the treatment of polytrauma by the DGU, are compared with practices observed in the field. These practices were evaluated retrospectively (post hoc) to assess the necessity for trauma team involvement. The comparison aims to identify and quantify both overtriage and undertriage in trauma care for geriatric patients.

Overtriage is defined as the activation of the trauma team without the presence of a criterion for high or moderate risk of injury and thus without any indication of the presence of a serious injury. Overtriage can detect injuries that were not initially expected, so that the patient benefits from it. The price for this is the use of personnel, material and conceptual resources. The patients are defined as “false positives”.

In contrast, with undertriage, no emergency room alarm is triggered despite the presence of a criterion for high or moderate risk of injury. This can result in a life-threatening delay in the care of the patient or even a complete lack of medical care. Undertriage is often linked to a lack of personnel, material and structural resources. These patients are defined as “false negatives”.

The post-hoc determined necessity for TTA is based on the TAcTIC (Trauma Team Activation and Trauma/Injury Care) consensus criteria (Table 1) [4]. The NIS (Emergency Medicine, Intensive Care, and Traumamanagement) Section of the DGU established the TAcTIC Study Group to optimize the care of severely injured patients, focusing on developing and implementing standardized trauma team activation protocols.

**Table 1:** Post-hoc criteria by Waydhas et al.(4) (TAcTIC section by NIS DGU):

Category	Criteria
*Injury severity*	Abbreviated injury scale (AIS) ≥ 4
*Intensive care unit (ICU)*	ICU admission (without intermediate care unit)
*ICU-length of stay*	ICU-length of stay > 24 h
*Mortality*	Death within 24 h
*Invasive procedures (pre-hospital or in the emergency room)*	Cardiopulmonary resuscitation
	Advanced airway management
	Chest tube or needle decompression
	Pericardiocentesis
	Tourniquet use (pre-hospital)
	Catecholamine administration
	Transfusion
	Surgical/therapeutic radiological intervention
*Abnormal vital signs*	Pulse oximetry (SpO2) < 90%
	Respiratory rate < 9 or > 29/min
	Systolic blood pressure < 90 mmHg
	Shock index > 0.9
	Glasgow Coma Scale (GCS) < 9
	Deterioration of GCS ≥ 2 points before admission
	Hypothermia < 35 °C

### Inclusion criteria

The study included all patients aged ≥ 18 years who sustained acute trauma and were admitted to the emergency department of one of the 12 participating hospitals via prehospital EMS within six hours of injury. The prehospital emergency team not necessarily included an emergency physician.

### Exclusion criteria

Exclusion criteria included self-admission, presentation to the emergency department via ambulance without prior prehospital emergency medical services involvement, absence of acute trauma, inter-hospital transfers, and secondary admissions.

Patients were screened for eligibility at the time of admission based on predefined inclusion and exclusion criteria. Therefore, cases that did not meet the inclusion criteria were excluded at admission. As a result, we do not have a documented count of all admissions that matched these exclusion criteria.

### Data collection

The study collected detailed data on several key variables to comprehensively describe the patient cohort and their clinical outcomes. The dataset included age, sex, trauma mechanism, pre- and in-hospital physiology, transportation characteristics (helicopter/ physician), trauma team activation (TTA), emergency interventions, emergency surgical interventions, ICU stay, and death within 48 h. Specific injury patterns were categorized according to the Abbreviated Injury Scale (AIS > 0) for the following regions: head, thorax, abdomen, spine, arms, legs, and pelvis. The cause of injury was categorized into violence, suicide attempt, or traffic accident and the mechanism (low) fall.

The data was documented pseudonymized in a web-based system hosted at the Institut für Notfallmedizin und Medizinmanagement (INM), LMU University Hospital in Munich. Data was checked for plausibility and completeness.

### Statistical analysis

The descriptive analysis included the number of patients and percentages for categorical data. For comparisons, the Chi-squared test was used for categorical data and Mann-Whitney U-test was used for metric data. A p-value < 0.05 was considered statistically significant. Statistical analyses were conducted using the SPSS software package (version 25, IBM Inc., Armonk, NY, USA).

## Results

### Patient demographics and trauma team activation

A total of 3,753 patients were included in the multicentre analysis, of whom 1,371 (36.5%) were classified as geriatric (≥ 70 years). The median age was 57 years across the cohort. For 974 patients (26.0%) trauma team was activated. TTA differed significantly by age group: 216 geriatric patients (15.8%) compared to 758 (31.8%) younger patients (< 70 years), indicating reduced TTA for older patients despite comparable injury severity.

**Table 2:** Comparison of characteristics between younger and older trauma patients

Characteristic	< 70 years	≥ 70 years	*p*-value
**Age Range in years (Median)**	18–69 (42)	70–104 (83)	-
**Number of Patients**	2,382	1,371	-
**Trauma Team Activation (TTA)**	758 (31,8%)	216 (15,8%)	< 0.001
**Male Gender**	1,569 (65,9%)	528 (38,5%)	< 0.001
**ASA 3/4 (322 missing values)**	169 (7,6%)	584 (48,2%)	< 0.001
**Injury Severity Score (ISS)**	Mean: 4,8 (SD 6,7) Median: 2 (IQR 1–5)	Mean: 5,5 (SD 6,0) Median: 4 (IQR 1–9)	< 0.001
**Injury Patterns (AIS > 0)**			
Head Injuries	816 (34,3%)	654 (47,7%)	< 0.001
Thoracic Injuries	329 (13,8%)	131 (9,6%)	< 0.001
Abdominal Injuries	84 (3,5%)	12 (0,9%)	< 0.001
Spinal Injuries	353 (14,8%)	149 (10,8%)	< 0.001
Upper Extremity Injuries	728 (30,6%)	299 (21,8%)	< 0.001
Lower Extremity Injuries	660 (27,7%)	403 (29,4%)	0.269
Pelvic Injuries	76 (3,2%)	78 (5,7%)	< 0.001
**Transport by helicopter**	247 (10,4%)	49 (3,6%)	< 0.001
**Transport with EP**	950 (39,9%)	334 (24,4%)	< 0.001
**Cause: Violence**	210 (8,9%)	3 (0,2%)	< 0.001
**Cause: Suicide**	43 (1,8%)	5 (0,4%)	< 0.001
**Cause: Traffic Accident**	805 (36,4%)	116 (8,5%)	< 0.001
**Mechanism: Fall**	847 (38,3%)	1,191 (87,3%)	< 0.001
**Hospital Admission**	1,242 (52,1%)	879 (64,1%)	< 0.001
**Intensive Care Treatment**	415 (17,4%)	251 (18,3%)	0.494
**Mortality (within 48 h)**	13 (0,5%)	25 (1,8%)	< 0.001

EP: Emergency medical service physician, ASA: American Society of Anesthesiologists Score, SD: Standard Deviation, IQR: Interquartile Range, p-value < 0.05 was considered statistically significant

### Injury profiles and severity scores

Table 2 emphasizes relevant differences in injury patterns, transport modalities, and outcomes between younger and older trauma patients. Geriatric patients were more likely to sustain head injuries (47.7% vs. 34.3%) and pelvic injuries (5.7% vs. 3.2%), while younger patients showed higher rates of thoracic (13.8% vs. 9.6%), spinal (14.8% vs. 10.9%), and upper extremity injuries (30.6% vs. 21.8%). Older patients also had higher rates of ASA 3/4 classification (48.2% vs. 7.6%), indicating a greater burden of preexisting comorbidities.

In terms of prehospital decisions, regarding transport and treatment, younger patients were more frequently transported by helicopter (10.4% vs. 3.6%) or accompanied by an emergency physician (39.9% vs. 24.4%).

The median Injury Severity Score (ISS) in geriatric patients was 4 (IQR: 1–9)points, slightly higher than the median ISS of 2 (IQR: 1–5) points observed in younger patients.

Cause and mechanism of injury differed markedly, with geriatric patients having fewer cases of trauma related to violence (0.2% vs. 8.9%) or traffic accidents (8.5% vs. 36.4%) but therefore substantially more (low) falls (87.3% vs. 38.3%).

Mortality within 48 h was notably higher in geriatric patients (1.8%) compared to younger ones (0.5%) Table 3. 

**Table 3:** Geriatric subgroup analysis (≥ 70 age Groups)

Age Group in years	*N*	Male gender	Mean ISS (Median)	48 h mortality
70–79	457	53%	6.09 (4)	1.1%
80–89	645	61.7%	5.39 (4)	2.2.%
> 90	269	75.5%	4.72 (2)	2.2%

### Subgroup analysis ≥ 70 years

Within the geriatric cohort, stratification by age decades revealed a decreasing trend in injury severity with increasing age. Patients aged 70–79 had the highest mean ISS (6.09), followed by those aged 80–89 (5.39) and 90+ (4.72). Similarly, the proportion of female patients decreased across age brackets, from 47.0% in the 70–79 group to 24.5% in patients aged 90 and above. Despite lower ISS values in the oldest subgroup, 48-hour mortality rates were slightly higher in the 80–89 and 90 + age groups (2.2%) compared to 1.1% in those aged 70–79.

### Post-Hoc analysis

Post-hoc analysis using the TAcTIC consensus criteria revealed that among the 1,371 geriatric patients, 1,083 (79%) did not meet any of the TAcTIC criteria, and 288 patients (21%) met at least one criterion for TTA. In 172 cases (12.5%), only a single criterion was met. Intensive care therapy was the most frequent criterion, observed in 62 cases (36.3%), followed by low oxygen saturation in 18.1% of cases.

**Fig. 1:**
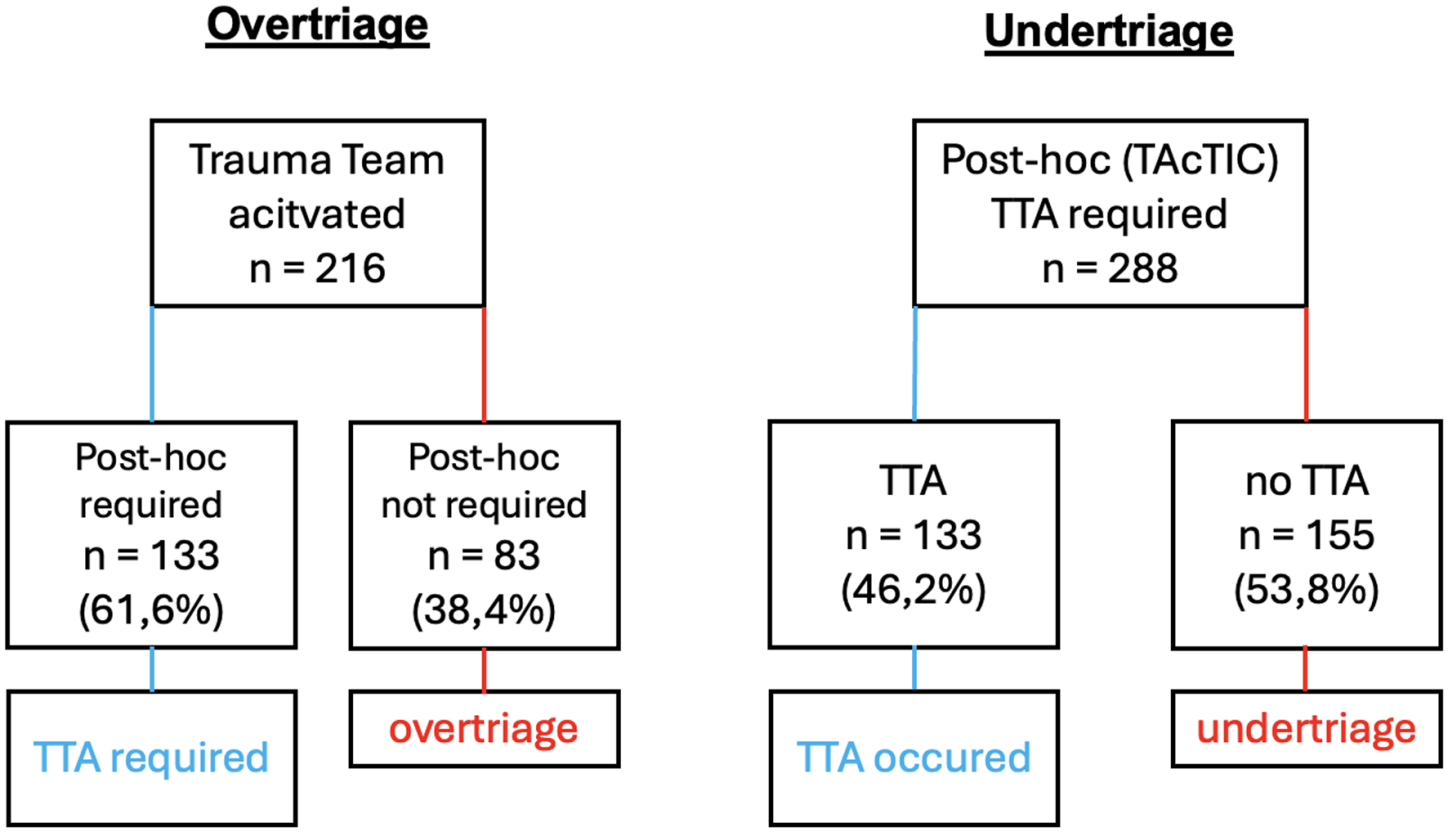
TAcTIC Trauma Team Activation: Over- and Undertriage in geriatric patients TTA = Trauma Team Activation, TAcTIC = Trauma Team Activation and Trauma/Injury Care

Of the 288 patients, only 133 (46.2%) were triaged for TTA. A substantial proportion, 155 cases (53.8%), were undertriaged, receiving no TTA despite meeting TAcTIC criteria. (Fig. 1)

Conversely, 83 geriatric patients (38.4%) who received TTA did not meet any TAcTIC criteria and were therefore preclinically overtriaged.

### Blood pressure < 100mmHg

In 862 cases the blood pressure was documented. 43 (5.0%) cases had systolic blood pressure < 100 mmHg. Among these, 37 cases (86.0%) required post-hoc specialized trauma care. TTA was noted in only 17 cases (39.5%), and 16 of these cases demonstrated post-hoc the necessity for TTA. There was an overtriage of 14% and undertriage of 56,8% (21 injured).

### Suspected traumatic brain injury (TBI) with GCS ≤ 14 points

Among 155 geriatric patients with prehospital GCS documentation, TTA was observed in 79 cases (51.0%), 66 of these cases confirmed as having post-hoc TTA requirement. In total 86 cases (55.5%) of 155 required post-hoc specialized trauma care. There was an overtriage of 59% and undertriage of 23,3%.

### Two or more injured body regions

A total of 144 patients (10.5%) were identified with injuries in two or more body regions with an AIS severity of ≥ 2. Of these, 74 cases (51.4%) required post-hoc specialized trauma care. Prehospital registration for TTA was recorded in 82 cases (56.9%), of which 60 cases met TTA criteria post-hoc. There was an overtriage of 48,6% and undertriage of 19%.

### Fracture of one or more long bones following a traffic accident

Among 38 cases (2.8%) with documented fractures of long bones, 14 cases (36.8%) required post-hoc specialized trauma care. Prehospital registration for TTA was noted in 23 cases (60.5%), with 12 cases confirmed as meeting TTA criteria post-hoc. There was an overtriage of 63,2% and undertriage of 14,3%.

Furthermore, in summary of Table 2, fewer injured geriatric patients are transported accompanied by an emergency physician. However, more geriatric patients require inpatient and/or high-care treatment than the comparison group of injured adults < 70 years. The criteria GCS ≤ 14 points, BP < 100mmHg and 2 + body regions (post-hoc) require an initial treatment in the majority of cases (undertriage!). The rate is particularly high in cases of low blood pressure (86%). The TTA requirement for fractures of the extremities is the lowest among the new criteria (37%), but is currently most frequently used as trauma team activation criterion (61%).

## Discussion

The undertriage of geriatric trauma patients remain a critical issue in emergency medical care, despite the implementation of structured trauma team activation (TTA) protocols. The existing guideline on “Polytrauma and Treatment of Severely Injured Patients” from 2023 by the DGU tried to capture the unique physiological vulnerabilities and atypical presentation patterns of geriatric patients. Nevertheless, the existing TTA criteria remain not sufficient enough, leading to a considerable rate of undertriage. Our study revealed an unique high rate of undertriage in 53.8% of cases, which can lead to delayed interventions and worse clinical outcomes. This finding is similar to evidence from the USA and Australia confirming a rise in undertriage with increasing age among older trauma patients [5, 6].

Recent studies emphasize that the physiological response to trauma in geriatric patients significantly differ from younger cohorts. Clare et al. [7] highlighted the blunted tachycardic response due to common medication use (e.g., beta-blockers), which can mask the severity of trauma in geriatric individuals. Furthermore, geriatric patients often have higher baseline blood pressures, rendering standard hypotension thresholds inadequate for this population. Among our patients with systolic blood pressure < 100 mmHg, 86% required specialized trauma care, yet only 39.5% received TTA, demonstrating a clear under-recognition of hemodynamic instability in this age group. As noted by Waydhas et al. [8], trauma team activation protocols that do not account for these differences may fail to identify critically injured geriatric patients in time, resulting in suboptimal care. Furthermore, Brown et al. [5] strongly recommended that a systolic blood pressure below 110 mmHg for geriatric trauma patients should be included as a Step 1 criterion in the National Trauma Triage Protocol (NTTP) in the USA.

In our findings geriatric patients represented 36.5% of the total cohort. TTA rates were significantly lower compared to younger patients (15.8% vs. 31.8%), despite comparable or even higher injury severity scores. This discrepancy indicates a clear gap in the current triage criteria, which fails to account for the unique needs of geriatric trauma patients. Multiple studies from the USA confirm our research results and identified that older trauma patients are significantly undertriaged and less likely to be transported to Trauma Centres (TCs) compared to younger patients [5, 9–11].

The discrepancy in prehospital transport decisions further illustrates potential biases in triage practices. Fewer geriatric patients were transported by helicopter (3.6% vs. 10.4% in younger patients) or accompanied by an emergency physician (24.4% vs. 39.9%). EMS physicians are common in the German-speaking area. However, geriatric patients demonstrated higher hospitalization rates (64.1% vs. 52.1%) and ICU admission rates (18.3% vs. 17.4%), emphasizing their greater need for intensive or high care despite reduced prehospital prioritization. This raise concerns that frailty, and age-related conditions may be underestimated by emergency medical services, with reduced general condition often misinterpreted as normal for this older population. This observation may indicate that EMS personnel prioritize younger patients for emergency physician-accompanied transport, assuming better outcomes with more aggressive interventions, while the complex needs of older patients are overlooked.

The TAcTIC consensus criteria revealed significant rates of undertriage in the post-hoc analysis [12]. Among the geriatric patients who met the criteria for TTA, more than half did not receive timely trauma team activation, emphasizing an insufficiency of the current protocols in addressing this population’s specific risks.

Moreover, specific injury patterns among the geriatric further complicate the triage process. Gioffrè-Florio et al. [13] and Koch et al. [14] found that head injuries, thoracic injuries and pelvic fractures are disproportionately more common in geriatric patients, yet these injuries often do not meet the activation criteria under current protocols. De Simone et al. [15] recommended that early trauma protocol activation for patients aged 55 years and older should include frailty assessments, comorbidities, and medication histories. Such recommendations align with our findings (ASA score 3 or 4: 48,2% vs. 7,6% for under 70 years), where undertriage was commonly observed in patients presenting with mild-to-moderate injury patterns that progressed to severe complications.

Our study found that the 48-hour mortality rate among geriatric trauma patients (1.8%) was more than three times higher than in younger patients (0.5%), emphasizing their increased vulnerability even after low-energy trauma. Undertriage, which results in the transport of severely injured patients to lower-level care facilities or not activating trauma team, has been recognized as a key factor in increased mortality among older adults in the United States and Sweden [16–18]. In combination with significantly higher incidence of severe traumatic brain injury and the lack of neurosurgical experience in low level trauma centres underscore this clinical problem with delayed decompressive cranial operations. Additionally, the issue of life threatening bleeding complications in geriatric patients with oral anticoagulation treatment in combination with sTBI or pelvic trauma should be mentioned. These studies show that treatment at designated trauma centres significantly reduce short- and long-term mortality (10.4% vs. 13.8%, 41% lower adjusted 30-day mortality), highlighting the importance of appropriate triage. On the other side higher mortality could be a result of underlying acute disease like pneumonia, stroke or heart diseases and therapy-limiting patients request. Therapy limitations such as do-not-resuscitate (DNR) or do-not-intubate (DNI) orders and restrictions on intensive interventions may have influenced mortality and undertriage rates. These limitations are frequently encountered in geriatric trauma care and can impact clinical decision-making. While respecting patient autonomy is essential, it is crucial to ensure that such factors do not lead to systematic undertreatment. Future studies should explore how therapy limitations affect triage decisions and outcomes.

Given the 53.8% under-triage rate observed in our study, it is likely that inadequate trauma team activation contributes to excess mortality, reinforcing the urgent need for revised triage protocols that better consider frailty and age-related physiological differences.

Another major barrier to appropriate trauma team activation for geriatric patients is the reliance on post-hoc ISS values, which inadequately reflect the acute care needs of this population. Waydhas et al. [19] demonstrated that many patients with ISS values below the common threshold of 16 still required life-saving interventions, emphasizing the need for criteria that incorporate vital signs and life-saving procedures directly, rather than only relying on anatomical injury scores. Bieler et al. 12) further stressed that ISS-based criteria often fail to capture frailty-related risks, which are critical determinants of outcomes in geriatric trauma patients.

Subgroup analysis of geriatric patients by decade (Table 2) showed decreasing injury severity with increasing age, while 48-hour mortality remained slightly higher in the oldest subgroups despite lower ISS. This may reflect a greater influence of frailty, comorbidities, or therapy limitations in the very old.

The implications of undertriage are severe and multifaceted. Geriatric trauma patients are more likely to experience prolonged hospital stays, complications, and higher mortality rates compared to younger patients with similar injury patterns. As demonstrated in our post-hoc analysis, a significant proportion of geriatric patients who required intensive care or were presented with abnormal vital signs did not receive trauma team activation. This oversight can result in delayed interventions, worsened patient outcomes, and increased healthcare costs.

### Limitations

The decisive reasons for activating a trauma team and who initiated it cannot be conclusively determined from our study data. The rate of false-negative suspected diagnoses concerning trauma triage criteria based on injury patterns remains unknown. Additionally, the study included only Level 1 trauma centres, which may lead to a selection bias in the patient population.

As we discussed, an additional important limitation is the lack of data regarding underlying or concurrent acute diseases or therapy-limiting patients requests among elderly patients. These factors may have contributed to the higher mortality rate observed in this population, as patients with documented therapy restrictions might have received less aggressive treatment. This aspect warrants further investigation and should be considered when interpreting our findings.

## Conclusion

Our study reveals a 53.8% undertriage rate in geriatric trauma patients, contributing to delayed trauma team activation and a threefold higher short-term mortality rate compared to younger patients. Current triage protocols often fail to consider frailty, comorbidities, and the altered physiological responses of geriatric patients, leading to inadequate trauma care.

To improve outcomes, adjustments to trauma team activation criteria are essential. Lowering physiological thresholds and incorporating frailty assessments into triage could enhance early identification of high-risk patients. Strengthening structured triage protocols and increasing awareness among emergency providers about the possible injury severity of low-energy trauma in the geriatric can further reduce undertriage.

An upcoming revision of the German S3-polytrauma guideline offers a crucial opportunity to integrate these changes. Future research should explore how therapy limitations, such as DNR/DNI orders, influence triage decisions to ensure frailty does not lead to undertreatment. Ultimately, adapting triage protocols to the aging population is critical to improving trauma care and survival outcomes for geriatric patients.

## Data Availability

The datasets generated and analysed during the current study are not publicly available due to ethical and legal restrictions but are available from the corresponding author upon reasonable request.

## Abbreviations

ACS COT: American College of Surgeons Committee on Trauma
AIS: Abbreviated Injury Scale
ATCN®: Advanced Trauma Care for Nurses
ATLS®: Advanced Trauma Life Support
ASA: American Society of Anesthesiologists Score
BP: Blood Pressure
CPR: Cardiopulmonary Resuscitation
DGU: German Trauma Society (Deutsche Gesellschaft für Unfallchirurgie)
DNR: Do Not Resuscitate
DNI: Do Not Intubate
EP: Emergency Physician
EMS: Emergency Medical Services
GCS: Glasgow Coma Scale
ICU: Intensive Care Unit
IQR: Interquartile Range
ISS: Injury Severity Score
NIS: Committee on Emergency Medicine, Intensive Care and Trauma Management (Sektion Notfall-, Intensivmedizin und Schwerverletztenversorgung)
NTTP: National Trauma Triage Protocol
PHTLS®: Prehospital Trauma Life Support
SD: Standard Deviation
SPSS: Statistical Package for the Social Sciences
TAcTIC: Trauma Team Activation and Trauma/Injury Care (Study Group by NIS)
TBI: Traumatic Brain Injury
TTA: Trauma Team Activation
